# Prevalence, antimicrobial susceptibilities and risk factors of Methicillin resistant *Staphylococcus aureus* (MRSA) in dairy bovines

**DOI:** 10.1186/s12917-022-03389-z

**Published:** 2022-07-29

**Authors:** Abdelfattah Selim, Khalid Kelis, Muneera D. F. AlKahtani, Fatima M. Albohairy, Kotb A. Attia

**Affiliations:** 1 Department of Animal Medicine (Infectious Diseases), Faculty of Veterinary Medicine, Benha University, Toukh, 13736 Egypt; 2 Internal Medicine/Adult Endocrinology/Bariatric Medicine, Adiriyah Hospital, MoH, Riyadh, Saudi Arabia; 3 Department of Biology, College of Science, Princess Nourah bint Abdulrahman University, P.O. Box 84428, Riyadh, 11671 Saudi Arabia; 4 Extramural Research Department, Health Sciences Research Center, Princess Nourah bint Abdulrahman University, P.O. Box 84428, Riyadh, 11671 Saudi Arabia; 5 Center of Excellence in Biotechnology Research, King Saud University, P.O. Box 2455, Riyadh, 11451 Saudi Arabia

**Keywords:** Methicillin resistant *Staphylococcus aureus*, Bovines, Phenotypically, Antibiotic susceptibility test, Risk factors, Egypt

## Abstract

*Staphylococcus aureus* is a common mastitis pathogen in dairy cows, and methicillin-resistant *S. aureus* (MRSA) has been found in dairy farms all over the world. The study carried out on bovines from three governorates in Egypt, with the goal of determining the prevalence of MRSA in positive milk samples of subclinical mastitis, performing an antibiotic susceptibility test against MRSA isolates and determining the risk factors associated with MRSA. A total of 350 quarter milk samples (*n* = 200 mixed breed cow; *n* = 150 water buffalo) were collected and examined for subclinical mastitis using the California mastitis test (CMT) before being exposed to standard microbiological procedures for *S. aureus* isolation. The disc diffusion method was used to phenotypically analyse the positive *S. aureus* isolates for MRSA, which was verified by a PCR assay targeting the *mecA* gene. According to the findings of the study, 41.4% (145/350) milk samples were positive based on CMT, while 35.7% (125/350) of positive samples identified as MRSA based on PCR assay. However, the obtained results revealed non-significant disparity between cattle and buffalo and all predicted risk factors were strongly associated with prevalence of subclinical mastitis. The in-vitro antibiotic susceptibility test revealed that cefoxitin was completely resistant, whereas linezolid, ciprofloxacin, and trimethoprim + sulphamethoxazole were sensitive against the MRSA isolates. The relevance of *S. aureus* to public health, as well as the development of resistance to antibiotics like methicillin, needs ongoing testing of antimicrobial medications against MRSA isolates.

## Introduction

Mastitis is a serious disease that affects bovines all over the world, resulting in significant economic losses due to intramammary infection in lactating animals [1, 2]. The dairy industry in Egypt is constantly exposed to a variety of health risks, the most common of which is mastitis, which is a major concern and risk factor in the sector’s development. It causes lower in milk output, milk condemnation, animal replacement, culling, and a drop in quarter-wise productivity [3, 4]. Overall, the frequency of mastitis pathogens varies greatly between herds and locations. Environmental *Streptococci* and *Coliform* bacteria appear to be the most prevalent pathogens causing clinical mastitis, followed by *Staphylococcus aureus* (*S. aureus)*. Despite the fact that treatment efforts are frequently limited, *S. aureus* infections are difficult to control and are well-known for generating subclinical, clinical, and chronic mastitis [5]. *S. aureus* has gained multidrug resistance, making it easier for it to infiltrate the immune system of the host [6]. Penicillin was thought to be effective against a wide range of *staphylococcal* infections, but by the mid-1940s, it had begun to develop resistance to *S. aureus* strains. Consequently, methicillin drugs (β-lactams antibiotics) have been developed, which are regarded effective against penicillin-resistant *S. aureus*. However, a *mecA* gene mediates the resistance of methicillin. This gene is found on a mobile genetic element called ‘‘*Staphylococcal* cassette chromosome mec’’ (SCCmec). The gene produces an altered penicillin-binding protein 2a (PBP2a). PBP2a has a weaker affinity for β-lactam antimicrobials than PBP. Accordingly, most of β-lactam antibiotics are ineffective against *mecA* /*mecC* positive *staphylococci*.

In Egypt, the unrestrained and massive use of antibiotics (particularly penicillin groups) for the treatment of bovines has resulted in the establishment of resistant *S. aureus* strains. *S. aureus’* methicillin resistance has a negative impact on the treatment of both animal and human diseases [7–10].

The prevalence of methicillin-resistant *S. aureus* (MRSA) was more common in the United States than in Europe [11]. According to various studies, prevalence rates in South Korea and Vietnam were over 70%, while prevalence rates in Portugal, Greece, and Italy were less than 50% [12]. According to a studies conducted in Egypt, the prevalence of *S. aureus* was 17.2% while 70–73% *S. aureus* strains obtained from a variety of foods were resistant to β-lactam antibiotics including penicillin and ampicillin [13]. Furthermore, bovines in the governorates of Dekahlia and Ismalia were found to have a high incidence of *S. aureus* strains isolated from subclinical mastitis and resistant to penicillin and cefoxitin [7, 14]. However, there have been few studies on the prevalence of *S. aureu*s and MRSA in dairy animals in Egypt, particularly in the study areas of the present study.

Therefore, the goal of this study was to determine the prevalence of subclinical mastitis, as well as MRSA, in relation to potential risk factors in some Egyptian governorates.

## Materials and methods

### Study area

The research was carried out in three governorates in northern Egypt in 2021: Beheira, Kafr ElSheikh, and Menofia. Geographically, these governorates are located at 30.61°N 30.43°E, 31°06′42′′N 30°56′45′′E, and 30.52°N 30.99°E, respectively. Egypt is mostly characterised by a hot desert environment (Köppen climate classification BWh). The yearly rainfall in the study region ranges from 200 to 400 mm, with a mean annual temperature of 25 °C. The study area’s agricultural production system is a mixed crop and livestock farming system.

### Sampling

The sample size was determined using Thrusfield’s formula [15], with a 95% confidence interval, a absolute precision of 5%, and a 50% predicted prevalence. A total of 350 dairy animals (200 mixed breed cattle and 150 water buffalo) raised by individual farmers, all of which appeared to be healthy, were chosen at random from the studied regions. Following the initial screening of subclinical mastitis (SCM) with the California Mastitis Test (CMT), milk samples were collected [16]. A total of 350 quarter milk samples (2 mL) were obtained from CMT positive cases and forwarded to Benha University’s Veterinary Diagnostic Laboratory in icebox for further analysis. A pre-defined questionnaire was used to collect information about risk factors such as species, age, hand washing between milking, parity, lactation stage, teat dipping, and mastitis history.

### Bacteriological examination

All of the milk samples (0.01 mL) were cultured on blood agar and incubated at 37 °C for 24–48 h. To confirm *S. aureus*, the bacterial colonies were streaked to Mannitol salt agar (Merck, Germany) and biochemically identified using Bergey’s Manual of Systematic Bacteriology’s recommended techniques [17]. Moreover, microscopy, Gram staining, and biochemical tests such as coagulase, catalase, and mannitol fermentation were used to investigate the presence of *S. aureus* [18].

### Phenotypic identification of MRSA and in-vitro antibiotic susceptibility test

To identify MRSA, 1 µg of oxacillin was applied to activate growth of *S. aureus* (0.5 McFarland) on Muller Hinton agar plates. The plates were incubated for 24 h at 37 °C. Vernier callipers were used to assess growth inhibition zones around antibiotic discs in comparison to standard zones as defined in the Clinical and Laboratory Standard Institute [19]. MRSA were defined as isolates resistant to oxacillin discs. According to the standard approach, antimicrobial susceptibility was determined using the Kirby-Bauer disc diffusion method [20]. The in-vitro antimicrobial susceptibility of MRSA to different antibiotics which was choose based on common antimicrobial treatments used in this region such as oxytetracycline (30 μg), ciprofloxacin (5 μg), gentamicin (10 μg), penicillin G (10 μg), ampicillin (10 μg), amikacin (30 μg), linezolid (30 μg), levofloxacin (5 μg), cefoxitin (30 μg), tylosin (30 μg), and trimethoprim + sulphamethoxazole (1.25 μg, 23.75 μg) was performed by putting antibiotic discs on Mueller Hinton agar streaked with activated MRSA growth of 1 × 10^8^ CFU/ml and incubated for 24 h at 37 °C. Vernier callipers were used to measure the growth inhibition zones surrounding the antibiotic discs, which were compared to CLSI 2020 criteria. The results were classified into three categories: sensitive, moderate, and resistant.

### Molecular detection of mecA gene

The *mecA* gene was confirmed by PCR in confirmed *S. aureus* samples that were resistant to oxacillin. The DNA was extracted using QIAamp DNA Mini (Qiagen, Hilden, Germany). The extracted DNA was examined using PCR assay targeting *mecA* gene with primers P1:5′-TGGCATTCGTGTCACAATCG-3′ and P2: 5′- CTGGAACTTGTTGAGCAGAG-3′ which amplify product of 310 bp, as described by Galdiero, et al. [21]. Identified *S. aureus* strain obtained from animal health research institute, Cairo was used as *mecA* positive control. The methicillin-resistant *S. aureus* isolates were confirmed by PCR assay in cattle (*n* = 73) and buffalo (*n* = 52).

### Statistical analysis

SPSS version 24 was used for the statistical analysis (SPSS Inc., Chicago, U.S.A.). The chi square test was used to assess the risk factors’ associations with the prevalence of SCM in a univariate analysis. Potential risk factors with a *P* value less than 0.2 were re-entered into the final model using multivariable logistic regression analysis. Risk variables with a *P* value of < 0.05 were determined to have a statistically significant link with positivity to SCM, and their confidence intervals (95% CI) and odds ratios (OR) were calculated.

## Results

The CMT found an overall prevalence of subclinical mastitis in bovines from the study areas of 41.4% (145/350). Cattle had a prevalence of 40.5% (81/200) versus 42.7% (64/150) in buffaloes. Moreover, the highest prevalence was observed in Kafr ElSheikh governorate in comparison with other localities, and it was 45.7% and 47.3% for cattle and buffalo, respectively (Table 1).

**Table 1:** Prevalence of *Staphylococcus aureus* in different localities

Locality	Cattle				Buffalo			
	**No of milk samples**	**No of positive (%)**	**95% CI**	***P*** value	**No of milk samples**	**No of positive (%)**	**95% CI**	***P*** value
Beheira	60	21 (35)	24.17–47.64	0.461	45	17 (37.8)	25.11–52.37	0.926
Kafr ElSheikh	70	32 (45.7)	34.57–57.3		55	26 (47.3)	34.69–60.21	
Menofia	70	28 (40)	29.33–51.71		50	21 (42)	29.38–55.77	
Total	200	81 (40.5)	33.94–47.42		150	64 (42.7)	35.04–50.67	

The resistance of *S. aureus* isolates to oxacillin was confirmed by PCR assay targeting the *mecA* gene. In the current study, MRSA was observed in 35.7% (125/350) of bovine milk from the governorates under the study.

Antibiotic sensitivity tests revealed that ciprofloxacin, and linezolid had 100% efficacy, levofloxacin had 85% efficacy, amikacin and trimethoprim + sulphamethoxazole had 80% efficacy, while tylosine, gentamicin, and oxytetracyline had 60%, 60%, and 40% efficacy against MRSA found in buffalo milk, respectively. Contrary, all MRSA isolates were resistant to cefoxitin. With a few exceptions, the efficacy of amikacin and trimethoprim + sulphamethoxazole was higher against MRSA isolates from cattle milk than that of buffalo milk, Table 2.

**Table 2:** Results of antimicrobial sensitivity test against Methicillin resistant *Staphylococcus aureus*

Antibiotic disc	potency	Cattle			Buffalo		
		**S**	**I**	**R**	**S**	**I**	**R**
Oxytetracycline	30 μg	40	–	60	40	20	40
Gentamicin	15 μg	60	–	40	60	10	30
Amikacin	30 μg	90	–	10	80	20	–
Ciprofloxcin	5 ug	100	–	–	100	–	–
Levofloxacin	5 μg	85	15	–	85	–	15
Linezolid	25 μg	100	–	–	100	–	–
Trimethoprim + Sulphamethoxazole	30 μg	100	–	–	80	10	10
Cefoxitin	30 μg	–	–	100	–	–	100
Tylosin	30 μg	60	20	20	60	20	20

*S* Sensitive, *R* Resistant, *I* Intermediate

The prevalence of subclinical mastitis was significantly higher (*P* < 0.05) in animals of median age group (> 4–8 years), particularly in animals in more than 4^th^ parity. Moreover, hygienic risk factors like hand washing between milking and teat dipping is significantly associated with prevalence of SCM. The lactation stage and history of previous mastitis were found significant predictor for occurrence of the disease, Table 3.

**Table 3:** Prevalence of subclinical mastitis in relation to different variables

Variable	level	No of samples	No of positive	%	95%CI	Statistic
Species	cattle	200	81	40.5	33.94–47.42	χ2 = 0.166 d = 1 *P* = 0.684
	buffalo	150	64	42.6	35.04–50.67	
Age (years)	2–4	80	28	35	25.45–45.92	χ2 = 11.643 d = 2 *P* = 0.003*
	> 4–8	158	81	51.3	43.54–58.94	
	> 8	112	36	32.1	24.21–41.26	
hand washing between milkings	Yes	140	28	20.00	14.22–27.39	χ2 = 44.155 d = 1 *P* < 0.0001*
	No	210	117	55.7	48.95–62.27	
parity	1–2	70	22	31.4	21.76–43.03	χ2 = 8.881 d = 2 *P* = 0.012
	3–4	180	70	38.9	32.07–46.17	
	> 4	100	53	53.00	43.29–62.49	
lactation stage	15–60	65	19	29.2	19.58–41.2	χ2 = 6.993 d = 2 *P* = 0.030*
	> 60–120	165	67	40.6	33.41–48.23	
	> 120	120	59	49.2	40.39–58	
Teat dipping	Yes	20	3	15.00	5.24–36.04	χ2 = 6.106 d = 1 *P* = 0.013*
	No	330	142	43.0	37.8–48.42	
history of previous mastitis	Yes	72	40	55.6	44.09–66.47	χ2 = 7.455 d = 1 *P* = 0.006*
	No	278	105	37.8	32.27–43.6	
Total		350	145	41.4	36.39–46.66	

^*^The result is significant at *P* < 0.05

A multivariate logistic regression analysis was used to investigate variables associated with the prevalence of SCM that were found in the univariate study. When compared to other animals, the risk of contracting the disease was shown to be 2.51, 2,65, 2,71, and 2.93 times higher in animals of median age (> 4–8 years), more than 4th parity, in lactation stage more than 120 days, and with a history of mastitis.Interestingly, the odds of disease were observed 6.56 and 4.86 times in absence of application of hand washing between milking and absence of teat dipping more than in case of application of hand washing or teat dipping, Table 4.

**Table 4:** Multivariable logistic regression analysis for risk factors associated with subclinical mastitis

Variable	B	S.E	OR	95% C.I. for OR		*P* value
				**Lower**	**Upper**	
Age (years)						
2–4	0.223	0.363	1.25	0.613	2.547	0.540
> 4–8	0.920	0.288	2.51	1.428	4.414	0.001
Hand washing between milkings						
No	1.881	0.286	6.56	3.745	11.503	< 0.0001
Parity						0.029
3–4	0.426	0.349	1.53	0.773	3.034	0.222
> 4	0.976	0.380	2.65	1.260	5.594	0.010
Lactation stage						
> 60–120	0.283	0.379	1.33	0.631	2.789	0.456
> 120	0.998	0.389	2.71	1.266	5.813	0.010
Teat dipping						
No	1.581	0.708	4.86	1.215	19.456	0.025
History of previous mastitis						
Yes	1.073	0.333	2.93	1.523	5.620	0.001

*B* Logistic regression coefficient, *SE* Standard error, *OR* Odds ratio, *CI* Confidence interval

## Discussion

The prevalence of antimicrobial resistance of *S. aureus* increased dramatically due to overuse of antibiotics, making treatment of clinical cases extremely difficult. Drug-resistant bacteria have arisen as a major public health concern, as these diseases can be transmitted to humans via animal byproducts [6, 22, 23].

In the present study, the overall prevalence of subclinical mastitis is directly in line with previous findings of Javed, et al. [24], it was 41.4% (145/350). Additionally, the prevalence of SCM was 40.5% and 42.6% in cattle and buffalo, respectively, which was higher in comparison to reported prevalence rate by Javed, et al. [24] in cattle and buffalo living in Pakistan.

The prevalence rate of MRSA was 35.7% (125/350) in the PCR assay targeting the *mec*A gene, which agrees with the findings of Aqib, et al. [25] and Abo-Shama [26], who identified 34% and 40% MRSA in bovine milk, respectively. However, the prevalence of MRSA was found to be lower in Michigan (0.6%) [27], Wisconsin (1.8%) [28], Germany (16.7%) [29], Korea (6.3%) [30], and India (13.1%) [31]. In terms of estimating MRSA prevalence, there are some inconsistencies. The lower prevalence of MRSA could be due to insufficient expression of the *mecA* gene or overproduction of beta-lactamase, which leads to disparity of diagnosis [32]. Moreover, the pH and osmolality of culture media, on the other hand, can affect phenotypic expression and the higher variety in MRSA strains can be a reason of diagnostic difficulty [33, 34].

In accordance with previous results of Javed, et al. [24] and Aqib, et al. [25], they observed all of MRSA strains are 100% sensitive to ciprofloxacin, moxifloxacin and linezolid but 100% resistant to cefoxitin. The current study’s findings on linezolid sensitivity and resistance to cefoxitin were similarly consistent with previous findings [35]. Also, the findings come in agreement with previous reports in Egypt, where most of *S. auraus* isolates are resist to oxytetracycline and cefoxitin [7, 14]. The results of this investigation contradicted those of Umaru, et al. [36], who found that 10% and 5% of MRSA isolates were resistant to sulphamethoxazole/trimethoprim and amikacin, respectively, while the findings of both studies were consistent in that ciprofloxacin was found to be sensitive against all isolates. However, no MRSA isolates from buffalo or cattle milk were found to be cefoxitin sensitive, which is consistent with Nemeghaire, et al. [35], who reported 100% resistance of MRSA isolates to cefoxitin.

From the results, it is clear that the prevalence of *S. aureus* increased significantly with age and number of parity. These findings are in accordance with findings of Elemo, et al. [37], who observed higher prevalence of *S. aureus* infection among older cattle with high number of calving. This could be due to pathogenic organisms being more susceptible in udders with relaxed sphincter muscles and the defence mechanisms of primiparous cows are more effective than those of multiparous cows [27].

Interestingly, unhygienic hands of milker are a possible source for disseminating of contagious pathogens during milking practices; these findings are consistent with prior findings [25].

The present findings found that the incidence of *S. aureus* varied greatly depending on the stage of lactation. When compared to early lactation, late lactation had a substantial effect on the prevalence of *S. aureus*. A similar conclusion was obtained by Abera, et al. [38] and Elemo, et al. [37]. These results could be attributable to chronic nature of mastitis, which is usually subclinical and more common in late stage of lactation. Moreover, *S. aureus* is the most common cause of subclinical mastitis [39].

Furthermore, in line of previous results of Abera, et al. [40], it can be concluded that animals having a history of mastitis exhibited a greater prevalence of *S. aureus* than animals without a history of mastitis. The obtained finding suggests that treating animals for mastitis may not be efficient in eliminating pathogens and those pathogens may be passed along from one lactation to the next.

The present results confirmed that teat dipping had significant effect on prevalence of *S. aureus*, which was consistent with research reported that washing of udder before milking reduce the spreading of *S. aureus*in lactating bovines [41, 42].

## Conclusion

The methicillin resistant *S. aureus* was prevalent in buffalo and cattle from all studied governorates, Egypt. The prevalence of *S. aureus in* lactating bovines was significantly associated with age, washing of hands between milking, number of parity and lactation, teat dipping and history of previous mastitis. Furthermore, ciprofloxacin, linezolid, and trimethoprim + sulphamethoxazole were found to be effective against MRSA isolates from cattle and buffalo in vitro. MRSA prevalence can be reduced through regular investigation and treatment protocols, proper surveillance, and the implementation of appropriate control measures.

## Data Availability

All data that were generated or analysed during this study are included in this published article.
